# Maggots in Medicine: A Narrative Review Discussing the Barriers to Maggot Debridement Therapy and Its Utilisation in the Treatment of Chronic Wounds

**DOI:** 10.3390/jcm13226746

**Published:** 2024-11-09

**Authors:** Zoe Mumford, Yamni Nigam

**Affiliations:** 1 Cardiac Intensive Therapy Unit, Morriston Hospital, Swansea SA6 6NL, UK; zmumford@btinternet.com; 2 Faculty of Medicine Health and Life Science, Swansea University, Swansea SA2 8PP, UK

**Keywords:** maggots, maggot debridement therapy, MDT, Lucilia, wound care

## Abstract

**Background**: There is currently no standardised guidance that supports any particular method of debridement. Maggot debridement therapy (MDT) is often used as a last-resort therapy over more conventional treatments, despite mounting evidence of its benefits. **Objectives**: This review aimed to critically analyse the systemic and individual barriers to MDT implementation and utilisation. As the primary providers of wound care, discussions are primarily focused on nursing care. **Search strategy**: The Preferred Reporting Items for Systematic Reviews and Meta-Analyses (PRISMA) was used to conduct a literature search of the studies published between 2012 and 2022 across four databases: CINAHL, Cochrane, British Nursing Index and PubMed. The keywords used for this search were based on the PICO (Population, Intervention, Comparison, Outcome) framework. Twenty-three main articles met the inclusion criteria. All the studies were quality appraised using a risk of bias tool and data were extracted using a predesigned form. The evidence base of the four main themes were discussed: (1) effectiveness of MDT compared to conventional treatments, (2) perceptions and stigma, (3) cost, training and accessibility and (4) side-effects. **Conclusions**: The findings of this review suggest that MDT is an underused and potentially very effective method of debridement compared to conventional treatments. The identified barriers could be mitigated with relatively low-cost solutions. More high-quality research is needed across all the barriers.

## 1. Introduction

A recent cohort study estimated the prevalence of chronic wounds to have increased by at least 71% in the last 10 years [1], with the cost of managing chronic wounds accounting for 6% of the total expenditure of the National Health Service (NHS) in Wales, averaging £1727 per patient [2]. Moreover, the humanistic cost on the physical and psychological health of people living with chronic wounds is vast, impacting mobility, employment, and quality of life [3]. Importantly, it is reported that the majority of chronic wounds are leg ulcers [4]. Therefore, it is vital that we critically analyse the evidence base that underpins current treatments and the management of chronic leg wounds within healthcare.

### 1.1. Hard-to-Heal Wounds

The timely management of chronic wounds is needed as these wounds often persist and the chances of successful wound closure decrease with wound age [5]. Chronic wounds are defined as wounds that fail to proceed through the normal phases of healing in an orderly manner and cannot restore their functional integrity within three months [6]. The most prevalent types include venous ulcers, arterial ulcers, diabetic ulcers and pressure ulcers [4]. The acronym, TIME (tissue, infection/inflammation, moisture balance and epithelial edge advancement) is a widely used structured framework that describes the four aspects of wound bed preparation needed for wound healing to take place [7]. This tool provides nurses and other clinicians with a systematic approach to treating and assessing chronic wounds [8].

In the UK, maggot debridement therapy (MDT) or ‘larval therapy’ is an available treatment for the debridement (removal of dead/unhealthy tissue) of chronic wounds, but evidence suggests it could also simultaneously target multiple aspects of the TIME framework and ultimately aid in healing [9]. Historically, military records have documented the positive effects of MDT in unintentionally infested wounds on the battlefield [10]. Today, there are several specialist laboratories worldwide that produce clinical-grade ‘medicinal maggots’ to treat chronic wounds.

However, the current National Institute for Health and Care Excellence guidelines [11] state that there is not enough high-quality evidence to support any particular method of debridement. Consequently, despite its potential benefits, MDT is often used as a last-resort therapy after more conventional treatments, such as surgical or autolytic debridement, have failed or are deemed not suitable [12]. Nurses are often the primary healthcare professionals involved in wound care, and they are required to deliver treatment and advice based on the best available evidence [13]. Therefore, with clear gaps in policy and guidelines, a review of the best available evidence comparing MDT to other therapies is needed to guide practice, ensuring nurses and other healthcare professionals (HCPs) are providing the best possible care to patients with chronic leg wounds.

### 1.2. What Is MDT?

MDT in the UK consists of placing a BioBag (aseptically reared larvae of *Lucilia sericata*, the green bottle fly), onto the wound bed for 3–7 days at a time and is thought to work via three mechanisms: debridement, disinfection and accelerated wound healing [14]. The larvae debride the wound by attaching to the wound bed and crawling around using their hooks. These larvae excrete proteolytic enzymes that break down non-viable tissue, which is then ingested [15]. The larval secretions may have further advantages by disrupting the bacterial biofilm common to chronic leg wounds, eliminating resistant strains of bacteria and aiding in disinfection [16]. Given the current global antibiotic resistance crisis, treatments that help reduce infection are vital in modern medicine. Finally, MDT may promote accelerated wound healing through potential positive effects on several stages of wound healing such as fibroblast migration, angiogenesis and growth factor production [17].

However, there is still limited high-quality evidence in the form of systematic reviews or randomised controlled trials on the effectiveness of MDT and other methods of chronic wound debridement. Consequently, there is no standardised guidance on when MDT may be the best choice of treatment [11]. This may pose a barrier to the utilisation of MDT in clinical practice and may partly be the reason why MDT is often used as a last resort [18].

There are a few reviews examining methods of debridement that have used systematic methodologies but due to the heterogeneity of the data, meta-analyses are not possible [15]. The one recent systematic review examining the mechanisms of MDT yielded very few studies (n = 5) and only included studies where hydrogel was the comparative intervention in the analysis, excluding surgical or sharp debridement [19]. In order to identify when MDT could be used as a first-line treatment, a comparison to a number of common interventions is needed. This is highlighted further by research showing how MDT could potentially be faster and more accurate than surgical debridement through exclusively targeting non-viable tissue [20].

Therefore, this review will critically analyse the current evidence base underpinning MDT as a method of debridement in chronic wounds compared to a number of conventional treatments. Additionally, we will evaluate/discuss the possible secondary beneficial mechanisms of MDT—disinfection and wound healing. We will also critically analyse the discrepancies between case studies, cohort studies and randomised controlled trials (RCTs) not explored in depth by previous reviews due to a focus of RCT data. For instance, longitudinal methodologies appear to yield more positive results for MDT than RCTs, possibly due to the fact that a longer follow-up time allows for the clinical observation of the entire healing process [21].

Importantly, despite the poor evidence base for any method of debridement in chronic wounds, MDT is still often considered a last resort therapy, which is indicative of the potential systemic and individual barriers to its utilisation that are not present in other methods of debridement. Three main themes/barriers to MDT implementation and utilisation emerged within an initial literature search. These included potential negative perceptions and stigma amongst nurses and patients, concerns over cost, training and accessibility to medicinal maggots and lastly, certain side-effects or contraindications of using live larvae.

Moreover, these barriers may serve to perpetuate a culture in healthcare where MDT is underutilised, which will only exacerbate recruitment issues, small sample sizes and the financial backing of robust studies on MDT [22]. Thus, an in-depth review of how systemic and individual barriers may prevent MDT being used and implemented is imperative to identify and discuss potential resolutions and future research needs. For ease of reading, therefore, we have divided our review into four parts: (1) evidence for the effectiveness of MDT (debridement, disinfection and wound healing), (2) MDT perceptions and stigma, (3) the cost, training and accessibility of MDT and (4) the side effects of MDT.

## 2. Method

### 2.1. Search Strategy

A comprehensive search (limited to English language studies between 2012 and 2022), using the advanced function across several databases, including, CINAHL, Cochrane, British Nursing Index and PubMed, was performed. Predominately nursing-orientated journals were chosen to ensure a clear focus to the specific field underpinning the research question. However, biomedical/medical databases were also explored using PubMed to encapsulate the literature specifically on the biological mechanisms of MDT.

The keywords used were based upon the Population, Intervention, Comparison and Outcome (PICO) framework [23]. The search terms used were (“maggot therapy” OR “maggot debridement therapy” OR “larval therapy” OR “larvae” OR “biodebridement” OR biosurgery OR biodebridement) AND (wound OR “leg ulcer*” OR “chronic wound*” OR “diabetic foot” OR “foot ulcer”). These terms were used in various combinations with the Boolean ‘OR’ and ‘AND’.

### 2.2. Selection of Studies

Studies were included in this literature review if they met the pre-defined criteria. For the purpose of this review, the Caldwell framework [24] for critiquing health research was used. This tool was designed to critique healthcare literature specifically and comprises a convenient scoring system lending itself to student nurse applications and independent reviewers [25].

#### Part 1. Evidence for the Effectiveness of MDT: Debridement, Disinfection and Wound Healing

There appears to be a dearth of high-quality evidence to support any particular method of debridement in chronic wounds [11]. Here, we critically analysed the evidence base surrounding the effectiveness of maggot debridement therapy (MDT) in the treatment of chronic wounds compared to three main treatments: autolytic, sharp and surgical debridement. Autolytic debridement (commonly known as hydrogel dressing) separates nonviable tissue from healthy tissue by encouraging the body’s own endogenous proteolytic enzymes. Sharp and surgical debridement involve the manual removal of non-viable tissue using a scalpel or blade. Sharp debridement can be performed by an appropriately trained individual, including nurses, but surgical debridement has to be performed in an operating theatre by a surgeon [26].

It is important to note that, there are other methods of debridement, such as the Debrisoft monofilament pad included in recent NICE guidelines [27]. However, due to the limited evidence base and randomised control trial (RCT) data that directly compares them to MDT, they were not included in this review. Additionally, whilst recent clinical trial data posits that free-range larvae may be significantly more effective than bagged larvae in debriding chronic wounds [28], the UK now only clinically provides for ‘the BioBag’ and so this review predominately focused on the use of bagged larvae.

### 2.3. Debridement

The debridement or removal of non-viable tissue is a crucial step in wound bed preparation needed for healing to take place [8]. Prior to selecting any method of debridement for chronic leg wounds, nurses and allied healthcare professionals need to still adopt a person-centred approach to care, performing a comprehensive assessment of the patient and the wound regardless.

Mudge and colleagues [29] conducted an RCT using 64 patients with venous and mixed arterial/venous leg ulcers, comparing MDT to hydrogel treatment. Their primary outcome measure was debridement, and a blinded assessor (unaware of intervention and control groups), was used to evaluate the debridement. The authors found that the percentage of ulcers debrided in the larval group of the study was significantly higher at 96.6% compared to 34.4% in the hydrogel group. They also found that statistically significant fewer dressing changes were needed in the MDT group to successfully debride compared to hydrogel [29]. This evidence may work in favour of MDT when nurses and allied HCPs are considering a cost–benefit analysis of selecting appropriate treatments. Whilst this study suggests MDT is considerably faster in debriding chronic wounds, the researchers also found that a significantly greater number of ulcers in the larval group resloughed within a fortnight compared to the control group [29]. However, in clinical practice, a maintenance dose of larvae may be recommended after debridement is achieved [30]. This was not used, as there was not a comparable maintenance dose for the hydrogel dressing to make a direct assessment.

Mudge and colleagues’ RCT data [29] suggest MDT can be an effective treatment in chronic leg wounds. However, a high attrition rate resulted in a smaller sample size, reducing the reliability of their findings. Nevertheless, other researchers [20], conducting a similar RCT with 105 venous ulcers using *L. sericata* larvae comparing MDT to hydrogel, reported that their study was subject to less participant dropout rates. This study also found that MDT was significantly faster than the control in the first week of treatment [20]. Another RCT comparing MDT and sharp debridement with a small sample size (n = 19) measured debridement as the increase in healthy granulation tissue [31]. They found that the median percentage of granulation tissue was 90% for the MDT group and only 60% for the control group. Findings from these three RCTs suggest that MDT may be considerably faster and more effective in terms of debriding chronic wounds compared to conventional treatments.

The above RCTs included power calculations not feasible in cohort/case study designs, reducing the risk of a type one error. Furthermore, all the RCTs were able to utilise blinded assessors to assess debridement to reduce the chance of observer bias confounding the results. However, even though RCTs may be considered one of the highest forms of evidence [32], there are still very limited studies directly comparing MDT to hydrogel, sharp and surgical debridement.

Though not as scientifically robust, a number of cohort studies have allowed for greater sample sizes and longer follow-up times. For example, Gilead and colleagues [33] conducted a cohort study with 435 patients using MDT and found that complete debridement was achieved in 82.1% of patients. Equally positive results were presented in a later cohort study on patients with diabetic lower limb ulcers [34]. This study showed that 98.5% of leg wounds were fully debrided with MDT and 90% achieved this in one week. Though the lack of a control group means direct comparisons to other treatments cannot be made [34], these large cohort studies still have some merit in demonstrating MDT’s effectiveness as a debridement therapy in chronic leg wounds.

Supporting the evidence discussed above, Siribumrungwong and colleagues conducted a systematic review where the primary outcome was the debridement of wounds of various aetiologies [35]. Ten studies were included, with MDT having a significantly higher rate of successful debridement than conventional treatments with low heterogeneity. Research would suggest that MDT could be a more effective method of debridement for chronic wounds than conventional treatments currently being used in the UK, such as hydrogel dressings. Therefore, MDT could be an effective, if not more effective, choice for first-line treatment in patients with multiple comorbid conditions that may not be suitable for treatments such as surgical debridement. However, the authors also concluded that MDT was slow in the face of surgical debridement, taking, on average, a few weeks to fully debride. Therefore, in the case of septic responses or invasive infections, initial surgical debridement may be more appropriate if immediate debridement is necessary.

### 2.4. Disinfection

Managing infection in chronic wounds is another pivotal step in wound bed preparation needed before healing can commence (Harries et al., 2016) [8]. Common species of bacteria found in chronic wound beds are *S. aureus* and *P. aeruginosa* [36]. Aggregates of bacteria are commonly encased in extracellular matrixes that make up a ‘biofilm’ on the surface of the wound. The biofilms present on chronic wounds as opposed to acute wounds are often polymicrobial and so may be resistant to standard antimicrobial treatments [37]. Therefore, even though MDT is primarily licenced for debridement it is important to critically analyse its potential disinfection properties.

A recent in vitro study [38] found that freeze-dried larval secretions with antibiotics (0.5%) were significantly effective in destroying biofilm stability for *S. aureus* and *P. aeruginosa*. Similar findings were obtained from in vivo studies. For example, two RCTs found that there was a higher number of infected ulcers in the control group (hydrogel) compared to MDT by the study’s completion [20,29]. However, neither effect was found to be statistically significant. It is possible that because disinfection was a secondary outcome of both of the studies, the data were not adequately powered to produce significant results.

Two other recent RCTs where disinfection was a primary outcome found that MDT significantly reduced the presence of infection compared to the control arm of the study for *P. aeruginosa*, *E. coli* and *S. aureus* [39,40]. This evidence suggests that MDT may have an antimicrobial effect on chronic wounds additional to debridement that other conventional therapies such as hydrogel do not. Case reports and cohort data show how this could ultimately improve patients’ quality of life and aid in chronic wound management by reducing the chance of amputation and the length of antibiotic treatment [33,34].

### 2.5. Wound Healing

There is evidence that MDT may accelerate wound healing through additional mechanisms to debridement. For example, a case study documenting MDT on a patient with critical limb ischaemia found that skin perfusion pressure on the dorsal and plantar aspects of the foot had increased after maggot treatment [41]. The authors surmised that MDT may contribute to increased blood supply to the wound, aiding healing. Furthermore, an in vitro study by Cazandar [42] demonstrates that maggot excretions/secretions may accelerate wound healing due to its effect on the body’s innate complement system. In chronic wounds, the inappropriate activation of this system can lead to prolonged inflammation; Cazandar and colleagues [42] showed how maggot excretions/secretions may reduce complement system activation, reducing inflammation that may be impeding on wound healing. Similarly, in vitro studies examining the potential wound healing properties of MDT have also suggested that maggot salivary glands increase cell metabolism and protein production that aid in fibroblast cell migration and the production of extracellular matrixes [43]. Fibroblasts play a critical role in wound healing, tissue formation and wound closure. Additionally, extensive research evidence has accumulated on the ability of maggots to increase blood vessel formation (angiogenesis), and therefore oxygen provision, to aid in new tissue formation in wounds following MDT treatment [44,45].

However, earlier in vivo randomised control data [20] found there was no significant difference in healing rates and time to wound closure between MDT and the control group or surgical and hydrogel treatment. A later RCT [31] also found no significant difference in wound healing rates between MDT and the control group or sharp debridement and silver dressings. Nevertheless, like the potential antimicrobial properties of MDT, there are limited RCTs that examine wound healing rates as a primary outcome of this study. Both of the RCTs above measured healing rates as a secondary outcome. Moreover, wound healing was measured via wound dimensions, whereas Harries and colleagues [8] suggest that a measurement of epithelial tissue may be a more accurate measure of healing.

Wilasrusmee [21] conducted a retrospective cohort study on 111 patients with diabetic lower limb ulcers. Healing was categorised as greater than 95% epithelial covering and the absence of a scab. They found that healing rates were seven times faster (19 weeks shorter) in the MDT group compared to conventional hydrogel therapy. This positive result may be due to the use of epithelial advancement as a measure of wound healing, but also the longer follow-up time not seen in randomised control trials. Therefore, the in vitro studies and limited cohort study data suggest that MDT can accelerate healing times in patients with lower limb ulcers compared to other conventional debridement treatments. However, more RTCs are needed that address the heterogeneity regarding the measurement of wound healing and longer follow-up times to fully understand the potential additional healing benefits of MDT.

Finally, the majority of the RCTs and cohort studies discussed above omitted compression therapy as part of the treatment despite it being the gold standard in the first-line treatment of leg ulcers [46]. Reinforcing this, an important randomised control trial showed that maggots in the MDT arm of the study were still able to successfully debride 84% of wounds by day four under 4-layer compression bandaging compared to the control which involved just compression therapy [47]. This suggests that larvae can still survive under compression bandaging and, so, high-quality research comparing MDT to other therapies where compression bandaging is included is needed. NICE guidelines [48] on venous leg ulcers state compression therapy should commence immediately as long as the ankle brachial pressure index is measured first to exclude arterial insufficiency.

Evidence would suggest, therefore, that MDT is effective in debriding chronic leg wounds and may be faster and more effective than conventional treatments such hydrogel dressings. However, more high-quality data are needed in order to influence policies regarding nurse and allied HCP decisions based on treatment selection. There is a particular lack of high-quality evidence directly comparing MDT to surgical or sharp debridement therapies. Having said that, nurses should consider the potential additional beneficial properties MDT may have in treating chronic leg wounds not seen in other treatments such as accelerated wound healing and disinfection. Future research should aim to address the heterogeneity in the measurement of debridement and wound healing and incorporate compression bandaging to reflect current treatment standards in the UK for venous leg ulcers.

#### Part 2. MDT Perceptions and Stigma

The utilisation of MDT is multifactorial and relies on a nurse/clinician and patient agreement. Early MDT literature has discussed the intrinsic dislike of maggots amongst the general public, dubbed the ‘Yuk factor’ [49], which may pose as an additional barrier to the acceptance and utilisation of MDT amongst patients and nursing staff. Importantly, as previously mentioned, nurses are required to recommend and provide treatment based on the best available evidence regardless of their own perceptions [13] to ensure the best possible patient outcomes [50]. Moreover, when selecting a debridement method, nurses should also consider the patient’s preferences and perceptions alongside clinical assessment of the wound [11]. Thus, it is imperative to critically discuss the patient/public and nursing perceptions and experiences of MDT and why these may pose a barrier to the utilisation of MDT in practice and potential resolutions.

### 2.6. Public and Patient Perceptions

Recently, a mixed-methods study examining public opinions and the perceptions of MDT as a treatment option for chronic wounds was conducted [51]. Qualitative data were initially collected from a small focus group where the identified themes were used to design a 22-item survey completed by 412 participants. More than a third of the participants felt that maggots were ‘disgusting’ and demonstrated a preference for more conventional forms of debridement/dressings if they were to have a chronic wound. Theorists suggest that disgust is a multifaceted emotion that has evolved primarily for protection [52]. These findings [51] are in line with the previously described ‘Yuk factor’ in the early MDT literature. Thus, it could be predicted that this negative emotion toward the use of larvae may be the prime reason behind their low uptake in healthcare [18]. However, this survey also showed that despite feelings of disgust, over a third of the participants would consider MDT as a first-line treatment, but most were more inclined to choose MDT with increased wound severity [51]. This reflects current clinical use within the NHS as MDT is largely considered to be a last resort therapy. As a result, there is no research looking at public perceptions of MDT as first-line treatment for chronic wounds. Thus, the hypothetical first-line treatment scenarios used in the above study are important in highlighting that, despite feelings of disgust, the public may be willing to try MDT as a first-line therapy. However, the use of hypothetical scenarios does not capture a number of the psychosocial factors that may influence health-related decisions of those actually living with chronic wounds. For example, McCaughan et al. [53] conducted semi-structured interviews using patients with chronic leg wounds through vascular clinics and community nurse referrals and found that they were in fact very accepting of MDT. There was still evidence of feelings of disgust among the patients akin to Nigam et al.’s survey [51], but the patients reported a strong willingness to try MDT due to the long-standing effects of having chronic wounds on their life. Moreover, research has shown that the patients who actually received MDT reported that any negative feelings toward MDT were outweighed by the satisfaction of care [29]. Thus, the described ‘Yuk factor’ may not be a barrier to the use of MDT in patients with chronic wounds in the face of psychosocial motivators as a result of living with long-standing wounds. Additionally, having knowledge of MDT and its possible positive benefits may increase the likelihood of choosing MDT as a first-line treatment.

Thorough interviews conducted in another detailed study [53] also emphasise the role of family as a social determinant and potential barrier to positive MDT perceptions and use. The patients reported family as positively impacting their decisions to try MDT and, similarly, the reverse was seen where a patient’s feelings of disgust and scepticism were reinforced by her husband. Moreover, these familial perceptions may pose a greater influence in those patients who may have to rely on support networks for the application of MDT in the community. Well-researched health determinant models outline the influence that social determinants such as family can have on health and health-related decisions [54]. Therefore, more qualitative research designs are needed to reveal more nuances of patient experiences of MDT [55]. Furthermore, health education focused on creating positive perceptions of MDT at the patient and public level are needed to ensure these perceptions do not pose a barrier to what potentially could be the best treatment for specific chronic leg wounds.

Other public health education initiatives in the adult population have shown how education through entertainment can change perceptions and motivate health improvements in the public [56]. Recently, the BBC medical drama *Casualty* featured episodes incorporating successful maggot treatments for patients with wounds. Following the airing of the MDT episodes, an analysis of pre- and post-exposure surveys showed that exposure to the MDT storyline increased awareness significantly across the 622 respondents, and that there was an increase in positive perceptions and acceptability in choosing MDT as a potential therapy for their own wound [57]. This study highlights the potential success of public engagement initiatives through different mediums such as entertainment and how they could have an impact on generating positive perceptions of MDT. Nevertheless, larger-scale longitudinal studies are needed to draw empirical conclusions on their design and successfulness.

So, whilst there is evidence to support feelings of disgust toward MDT among the general public, public education at the patient and public level that disseminate facts, and the potential benefits of MDT could override these negative emotions and increase acceptance and uptake amongst patients and their support networks.

However, the utilisation of MDT is a multifactorial nurse/clinician and patient agreement. Nurses are often the most patient-facing and, in a separate study, how MDT was presented to patients, if at all, was reported as another important factor in MDT acceptance [53]. Thus, it is also important to discuss nurse and healthcare professional perceptions of MDT as a potential barrier to its implementation and utilisation.

### 2.7. Nurse’s Perceptions

Nurses in the UK are often leaders in the management of MDT both in hospital and community settings. Therefore, they are uniquely placed to be facilitators of nurse–patient communication regarding the potential benefits of MDT in specific cases and to effectively perform the application of the therapy itself. Pajarillo et al. [58] conducted a qualitative study using semi-structured phone interviews to examine healthcare professionals’ (HCPs) perceptions of MDT. Firstly, they found there to be potential barriers at the organisational level regarding the use of MDT, as there was reported misinformation/stigma about disease transmission, and further accurate information was needed to be sought from medical maggot companies before hospital approval. Secondly, disgust was reported as the major barrier to HCP acceptance and arguably shown to be stronger in nurses, as they reported being more ‘hands on’ with the treatment. Interestingly, most participants reported a positive attitude toward MDT but explained that they had observed these barriers amongst their colleagues [58]. This is a likely an example of the ‘Hawthorne effect’ impeding the internal validity of this study design, where participants modified their answers to reflect positively about themselves because they were part of the study. In a similar vein, in a study with a face-to-face interview design investigating nurse perceptions from 30 plastic surgical wards in Dublin, the researchers were able to see non-verbal signs of disgust such as wincing in response to questions on leech therapy [59]. Moreover, nurses reported being deterred from leech therapy as they had concerns about not being able to hide their own discomfort in front of the patients. Thus, there seems to be some evidence of disgust towards the therapy’s use of live organisms, not just in the general public, but among nurses and allied HCPs. Any misinformation/stigma at the organisational level could also potentially prevent the use of such therapies, and in the case of MDT, authors suggest a need for emotional preparation incorporated into MDT education for nurses to combat this [58].

Whilst Pajarillo et al.’s interviews [58] used a small sample size with only two nurses amongst the other HCPs, a more recent study by Hopkins et al. [60] used a mixed-methods design which included a survey of 160 nurses and 12 semi-structured interviews examining the lived experiences of MDT amongst nurses in the UK. Within the survey, a significant number of non-wound specialist nurses said they would consider MDT as a first-line treatment. In contrast to previous studies, even though disgust was verbalised by most of the nurses in the interview section, some of the nurses demonstrated positive attitudes towards the therapy and visible non-verbal ques of enthusiasm were reported by the authors, such as ‘animated’ facial expressions. This is a prime example of where face-to-face qualitative data can be imperative to understanding the nuances of lived experience [60]. Similarly, Bazalinski et al. [61] looked at the readiness of nurses to use MDT in practice and found that feelings of disgust, although reported, were not necessarily enough to deter staff from choosing MDT. Thus, both larger scale quantitative data and qualitative responses suggest positive attitudes towards MDT use amongst nurses. Yet, MDT is still primarily used as a last resort therapy for chronic leg wounds [18].

It may be useful to understand the perceptions and acceptance of MDT amongst nurses through Ajzen’s ‘Theory of Planned Behaviour’ [62]. This well-documented theory posits that the intention to recommend or chose MDT is guided by more than just positive attitudes but the subjective norms (societal and organisational norms) and the perceived behavioural control (self-efficacy, perceived knowledge and competency) surrounding MDT as well. The survey findings from Hopkins et al.’s study [60] could be seen to support this theory. For instance, 94.8% of non-wound specialist nurses reported considerable uncertainty in their own knowledge and confidence to use and recommend MDT, an example of where limited perceived behavioural control is a barrier to utilisation. Additionally, 57.1% of the nurses reported the lack of support and willingness of other staff to use and recommend MDT, which are potential subjective/organisational norm barriers [60].

These results give us valuable insight into the different barriers between nurses and MDT use/implementation. Hopkins et al.’s results [60] also emphasise the need for education on the mechanisms, benefits and application of MDT within the nursing curriculum, as research suggests that the level at which it is currently taught in the UK is limited, if present at all [60]. Furthermore, wound specialist nurses who have seen the positive effects of MDT and have a greater understanding were more likely to embrace MDT as a treatment option. Here, we can apply Rogers’s ‘Diffusion Theory’ [63] that describes how new practises can be spread throughout a population. This theory would emphasise the importance of newly qualified nurses (educated on MDT) and wound specialist nurses with positive experiences of MDT as key ‘innovators’ or ‘early adopters’. That is, organisations within the NHS should identify key individuals who are open and interested or already knowledgeable in MDT to be horizontal influencers and peer educators within their own healthcare settings. Research would suggest that this may help to increase self-efficacy to perform MDT alongside its inclusion in the nursing curriculum and aid a social norm that supports the use of MDT within healthcare. More empirical research on MDT effectiveness is also needed to dispel some existing counterintuitive thinking that MDT is an ‘outdated’ therapy [58].

There is some recent evidence emerging that suggests that nurse training/education on the mechanisms and benefits of MDT can have a positive effect on their readiness to apply MDT. Bazalinski et al. [61] surveyed 290 nurses in Poland and found a positive effect of nursing education and vocational training on the readiness to apply MDT, but also nurses’ perceptions of MDT itself. However, these conclusions are based on correlations and so we cannot infer direct causation as there may still be a number of mediating factors. The authors do acknowledge that ‘disgust’ towards MDT may still be an influential factor in nurses’ readiness as well, and, in a later publication [64], the research group found that following a visual perception exercise, the selection of pictures of maggots on a wound as the most repulsive image was associated with a personal appraisal of not being ready to implement maggot therapy. Neither gender nor perceived stress level, however, were exclusively associated with disgust for maggots or the lack of readiness to implement MDT [64]. The authors concluded that low professional experience and a deficit of knowledge in maggot therapy impacted negatively on the readiness of nurses to administer the therapy. Having said that, the benefits of MDT knowledge/education found in the earlier study [61] are in line with previously discussed findings [60] that show that wound specialist nurses with an increased knowledge of MDT are more likely to recommend and have positive attitudes towards the therapy. Thus, positive attitudes in nurses could be enhanced by introducing MDT education at various levels of the curriculum, including both undergraduate and postgraduate, as well as in in-hospital training. There is some American literature that begins to standardise MDT education into six domains designed to not only inform nurses but ensure they are safe and competent enough to recommend and perform MDT [65]. Therefore, the consideration of standardising MDT education and its implementation into UK curriculums is proposed, as it may not only increase the likelihood of MDT being used but, in line with the Nursing and Midwifery Council’s code of practice [13], it should optimise the delivery/quality of patient care by ensuring nurses have the knowledge and skills to practice safely and effectively.

Other barriers to MDT, such as cost/funding, have also been highlighted within the literature evaluated so far. Whilst American studies emphasise the lack of funding or insurance coverage surrounding MDT [61], UK studies highlight that accessibility to medicinal maggots could be a more notable barrier [60]. Wound specialist nurses who have positive attitudes and the perceived competency to perform MDT as well as team support would theoretically be indicative of the intention to recommend MDT [62]. However, some evidence reports that they are unlikely to utilise MDT because of the problems with ordering and dispensing medicinal maggots in the first instance [60].

#### Part 3. Cost, Training and Accessibility of MDT

The financial pressures on the NHS are widely known and so the national guidelines state that treatments with the lowest acquisition cost appropriate to the clinical circumstances should be used [27]. Here, we critically discuss how the cost, training and accessibility of MDT may be a barrier to its utilisation and implementation.

### 2.8. Cost

There is limited UK-based high-quality data regarding the cost–benefit analysis of MDT compared to other treatments. Nurses and allied HCPs are a major driver in the choice of debridement and so qualitative data on the perceived barriers to the decision to use MDT are important. For instance, qualitative interviews and survey data have found that even though nurses deemed MDT cost-effective in comparison to the mass order of hydrogel (autolytic) dressings ordered that may not be used, cost was still a predominant barrier to its utilisation by wound specialist nurses [60]. The current literature search only yielded one UK study where the cost of debridement was a primary outcome. Bennett and colleagues [66] compared the cost of MDT to other conventional treatments by calculating their cost ratios which attempted to consider both the costs incurred along the treatment timeline and the benefits gained. Benefits were measured as quality-adjusted life years (QALYs) derived from therapy utility, the likelihood of infection, the need for clinical interventions (e.g., amputation) and other adverse events. The authors concluded that surgical and sharp therapies were very high-cost compared to MDT and hydrogel dressings. Furthermore, even though the base cost of MDT could be more expensive per month or procedure (hydrogel = £246.67, MDT = £571.31), a cost–utility analysis suggested that MDT may be more cost-effective when considering money per QUALYs compared to hydrogel.

Due to limited evidence on cost in debridement, Bennett and colleagues relied somewhat on expert opinion to formulate their analysis [66]. Considered one of the lowest forms of evidence in terms of scientific rigour [32], caution should therefore be applied to their findings. A more recent bibliography found considerable heterogeneity in its cost–analysis with some studies favouring MDT and others, hydrogel [12]. Studies with longer follow-up periods that included accumulated costs of dressing changes and the treatment of infection found that MDT was roughly half the cost of hydrogel dressings [21]. These findings may be due to the faster debridement time in the MDT group. Similarly, Mudge and colleague’s RCT found that, even though cost was not a primary outcome, a significant increase in the dressing changes were needed in the hydrogel arm compared to MDT (*p* < 0.001) [29]. Therefore, research suggests that cost should not necessarily pose a barrier to MDT’s utilisation as it fares similar to hydrogel if not better when considering the amount of time for debridement and adverse events. However, there is considerable heterogeneity in the data, possibly due to comparing across healthcare systems with different financial pressures and providers of larvae.

### 2.9. Training

The evidence discussed so far regarding the cost of MDT and other debridement treatments mostly considers the additional cost of staff/specialist staff required to carry out the debridement technique such as surgeons for surgical debridement. However, there is limited data on the additional cost of training staff and how that would factor into cost–benefit/utility analyses. Unlike surgical and sharp debridement which require specialist input, MDT and autolytic (hydrogel) debridement can be performed by generalists with basic training. Wound management is predominantly nurse-led, and it is an integral part of a nurse’s role to keep their knowledge and skills up to date through regular learning and professional activities [13]. Therefore, informative packages on MDT incorporated into the mandatory staff e-learning within the NHS would incur minimal additional cost. The All Wales Tissue Viability Nurse forum guidance on MDT [14] could be used along with other existing online training tools such as NHS Education for Scotland’s’ LearnPRO [67], which explores debridement methods and when they are indicated.

Whilst a relevant systematic review suggests that e-learning can be as effective as traditional face-to-face learning [68], trusts may want to consider evidence-based standardisation to ensure the effective delivery of MDT so as not to compromise patient care. For example, a recent American study outlines key qualities and minimum standards needed for HCPs to competently perform MDT that could be used to map onto training initiatives in the UK [65]. Because MDT can be performed by generalists with basic training, this would suggest a low additional cost. Furthermore, the incorporation of MDT mechanisms and benefits into online learning packages may serve to act on two barriers in MDT utilisation by minimising cost but also reducing the stigma or ‘Yuk factor’ amongst nurses that may be preventing the use of MDT in healthcare. After all, studies have shown that specialist nurses in the UK who have increased knowledge of MDT have more positive attitudes towards it and are more likely to recommend the treatment than non-specialist nurses [60]. In addition, as stated in the ‘Effective debridement in a changing NHS UK consensus’ [69], alongside educational packages, organisations must make sure staff are aware of how to access specialist tissue–viability/wound support.

### 2.10. Accessibility

There are currently only a few producers of medical-grade maggots worldwide and only one supplier in the UK. Qualitative data from a UK-based study reveal that acute and community-based nurses are reluctant to order larvae because of ordering and dispensing difficulties, dubbed ‘long-winded’ and ‘unclear’ [60]. BioMonde (UK provider, Bridgend, UK) has recently addressed some of these issues by ensuring that orders placed by 2 pm will have a delivery early the following day (BioMonde personal communication).

Nevertheless, the evidence base discussed so far would suggest that if certain barriers are addressed, the demand for MDT may increase, potentially beyond the capacity of the current UK provider. Further exploration is needed on all echelons of the MDT supply chain to examine how an increase in demand could successfully be met. For example, Stadler conducted a review on the supply chain of MDT in Australia [70] and noted that even though it is known that live larvae are highly perishable and need to be applied to the wound within 24 h [71], there is limited literature regarding distribution and transport logistics. Other researchers discuss potential solutions such as finding ways for successfully storing eggs and pupae for extended periods to provide a buffer inventory to cope with spikes in demand [72], and others suggest that existing cool/cold chain solutions, used already to safely distribute perishable goods such as vaccines, could be used to inform future research on MDT distribution logistics [73]. However, ultimately, barriers to MDT utilisation feed into each other. Lack of awareness or negative stigma will be major barriers to MDT uptake and supply chain management. Additionally, high quality RCTs on MDT effectiveness and qualitative data regarding user experience will also serve to strengthen supply chain performance. Thus, a holistic approach to the barriers to MDT utilisation is imperative.

#### Part 4. Side Effects of MDT

Nurses require a professional duty of candour, whereby they must use current evidence to reduce the chance of a treatment causing harm to patients; including being open and honest with patients regarding any potential risks prior to treatment selection [13].

It is therefore important to note that there are certain risks common to most methods of debridement; for example, the use of MDT, autolytic (hydrogel), sharp and surgical debridement is contraindicated in wounds close to major blood vessels/arterial structures [14]. Thus, regardless of the method of debridement, nurses and allied healthcare professionals should ensure referral to tissue viability and/or podiatry and await the results of the recommended vascular studies stated within their individual trust policy before recommending or commencing treatment. There are, however, certain risks that may be more associated with MDT and, so, an analysis of the literature is needed to assess to what extent these should impede the implementation over other treatments.

### 2.11. Infection

Whilst MDT may aid in the disinfection of a wound through a variety of mechanisms discussed earlier, some case studies suggest that MDT may actually increase the risk of infection. For instance, Bueide and colleagues documented a case where a 70-year-old male’s wound treated with MDT resulted in Wohlfahrtiimonas infection [74]. Importantly, the use of maggots in medicine is not regulated in most countries and these particular maggots were sought through an unregulated naturopathic provider [74]. In the UK, however, infection is far less likely, as there are strict regulations and testing on aseptically produced clinical-grade maggots. Some studies do, however, show that the potential anti-microbial effects of medicinal maggots may be resistant to some Gram-negative organisms (e.g., *P. aeruginosa*) and so it may actually increase the presence of *P. aeruginosa* across the wound bed potentially due to a decrease in competition from other microbes [20,75]. However, this should not necessarily pose a barrier to MDT utilisation, as other methods of debridement such as hydrogel can also increase the risk of infection due to the high concentration of moisture, making the wound an ideal environment for bacterial growth [76]. In fact, a review of RCTs found no difference in the risk of infection between hydrogel (autolytic) debridement and MDT and in some cases, a greater decrease in infection rates was found in the MDT group [77].

### 2.12. Pain

Pain is a well-documented side-effect of MDT. Mudge et al. [29] conducted an RCT reviewing the participants’ perceptions of pain every three to four days during MDT until debridement was complete or up to 21 days. They found that for both the hydrogel and MDT groups, there was a statistically significant reduction in pain by the last appointment. In line with current MDT guidance [30], this would suggest MDT-associated pain usually manifests within the first few days of treatment but should improve over time. Therefore, akin to sharp debridement, nurses may want to consider pre-emptive analgesia when applying MDT, especially in those with known chronic pain exacerbations [18]. In the randomised control trial by Opletalova and colleagues [20], pain scores remained low over 15 days of MDT whilst controlling for neuropathy. Whilst pain was the primary reason for participant withdrawal in this RCT, qualitative data shows that patients who stopped MDT early maintained that the larvae had benefitted their ulcer and in those who continued with treatment, the pain was outweighed by the satisfaction of their care [29,53]. Thus, research would suggest that the pain experienced by MDT is relatively mild, with potentially worse perceptions of pain at the start of treatment. Importantly, there are a number of different biopsychosocial influencers that can affect a person’s perceptions of pain: the physical nature of the pain, emotions/anxiety, beliefs and attitudes and past experiences [78]. In the two RCTs described earlier [20,29], numerical visual analogue scales were used to rate pain which provided limited understanding as to the pain experienced during MDT. For example, researchers found that when patients were blindfolded during dressing changes and unaware of whether they were receiving MDT or surgical debridement [20], the thought of receiving live maggots was enough for a number of the participants in both of the groups to report a sensation of ‘crawling’ discomfort. Thus, the reported pain sensations are highly subjective. Future research could perhaps utilise holistic pain assessment tools, allowing for a better understanding of how large a role the psychological component of pain plays in the actual pain experienced in MDT. Research should also include an analysis of the effectiveness of pharmacological and/or educational nurse-led interventions on mitigating pain [51]. Lastly, pain is predominately a secondary outcome measure amongst the literature resulting in smaller sample sizes and limited statistical power, and so the estimation of pain before, during and post-MDT should be treated with caution. More research is needed where a holistic measure of pain is used and where pain is the primary outcome measure to better understand how nurses can manage potential pain exacerbations in MDT. It is important to acknowledge new research surrounding the potential side-effects of MDT that may not yet be reflected in current treatment guidelines and policy. For example, a recent case study details a 44-year-old man’s experience of MDT in an infected ulcer on a below-knee amputation site [79]. This patient had previous phantom limb pain that had subsided. On day two of MDT, he reported unbearable pain characteristic of the previously experienced phantom limb pain. This could be explained by the sensitisation or stimulation of the nerve fibres as a result of one of the potential secondary mechanisms hypothesised for MDT—nerve regeneration [80]. Thus, though not specifically stated in the ‘All Wales’ MDT guidance [14], MDT could be considered contraindicated in amputated limb sites. Whilst case report findings are generally considered to be restrictive in terms of generalisability and replicability [81], the best available evidence examining MDT in amputated limbs is currently limited to single case studies. Therefore, nurses should be aware of the newly documented experiences of MDT and in this particular case, should be cautious when considering MDT in amputated limb sites; it is important to explain the possible risk of recurrent phantom limb pain to patients to ensure they can make an informed choice.

In conclusion, research would suggest that the risks and side-effects of MDT are relatively minimal and, in most instances, can be pre-empted and prevented. Nurses and allied HCPs could play a vital role in mitigating these risks by carrying out a holistic assessment of the patient’s suitability for MDT, by following MDT utilisation guidelines and by being aware of new research regarding other potential contraindications and side-effects. If in any doubt, HCPs should contact the supplier of the medicinal maggots.

## 3. Conclusions

This review has examined multiple barriers to the utilisation and implementation of maggot debridement therapy in the treatment of hard-to-heal leg wounds. Firstly, evidence would suggest that MDT can be an effective first-line treatment in the debridement of chronic leg wounds and may be faster than conventional treatments such as hydrogel. However, more high-quality RCTs are needed to inform policy, specifically comparing MDT to surgical and sharp debridement methods. Importantly, nurses need to perhaps consider the evidence for the potential additional benefits of MDT over and above conventional treatments such as disinfection and accelerated wound healing.

Secondly, this review highlights that public and nurse perceptions of MDT can act as barriers to the use of MDT in healthcare. Education initiatives at the micro (patient/nurse) and macro (public) level to combat this barrier were discussed. NHS trusts could consider the use of key individuals, such as wound specialist nurses, who could increase positive awareness as horizontal influencers and peer educators within their own healthcare settings.

Thirdly, evidence would suggest that cost should not necessarily act as a barrier to MDT use, as it appears more cost-effective than surgical and sharp debridement and fares similar to hydrogel if not better when considering the amount of time to debride and adverse events. Furthermore, UK suppliers of medicinal maggots have supposedly improved their supply and demand turnaround times to improve accessibility. Existing cool/cold chain solutions for the distribution of other perishable goods could be used to inform future research on MDT distribution logistics if demand were to increase.

Lastly, the side-effects of MDT are relatively minimal compared to other conventional debriding treatments and can often be pre-empted and prevented by nursing staff if a comprehensive assessment of the patient is undertaken. Moreover, qualitative data reveal that any discomfort experienced is often outweighed by the satisfaction of the treatment outcomes.

Notably, this review uniquely identifies how the barriers to using MDT in healthcare interact with one another. For instance, a lack of awareness and negative stigma amongst the public and nurses will be a major barrier to MDT uptake and supply chain management, increasing cost and limiting accessibility. Similarly, high-quality RCTs on MDT effectiveness and qualitative data regarding user experience may strengthen supply chain performance and reduce negative perceptions. Thus, strategies to address these barriers should take a holistic approach and aim to address multiple aspects simultaneously where possible.

The findings from this review can be used by nurses and allied healthcare professionals as a summary of the best available evidence regarding the effectiveness of MDT compared to other conventional methods of debridement and potential barriers that need to be addressed. With no formal guidance to support any method of debridement, HCPs should still ensure that practice is evidence-based to achieve the best possible patient outcomes. Therefore, nurses should perhaps better educate themselves on the literature discussed in this review to ensure that where MDT may be the most suitable treatment for a patient’s chronic leg wound that it is in fact used and used appropriately.

Despite all of the literature discussed here, it is still evident that there is a need for more high-quality research across all the barriers discussed in order to effectively influence policy and guidelines for nurses as to the utilisation and implementation of MDT.
